# Supplementary material for: Induction chemotherapy followed by definitive chemoradiotherapy versus chemoradiotherapy alone in esophageal squamous cell carcinoma: a randomized phase II trial
Source: Nat Commun. 2021 Jun 29;12:4014. doi: 10.1038/s41467-021-24288-1 (PMC8242031; doi:10.1038/s41467-021-24288-1)

Supplementary Material

Number of supplementary Tables: 4
Number of supplementary Figures: 2

Supplementary Table 1. Compliance to induction chemotherapy
Supplementary Table 2. Compliance to concurrent chemoradiotherapy
Supplementary Table 3. Death reason according to treatment group
Supplementary Table 4. Adverse events during induction chemotherapy
Supplementary Figure 1. Compliance to concurrent chemotherapy (A) and actual radiation dose (B).
Supplementary Figure 2. Cumulative incidence of recurrences in the two treatment groups.

Supplementary Table 1. Compliance to induction chemotherapy

Variable	IC + CRT Group (n = 55)
Patients completing two cycles of IC	55 (100%)
Patients with dose reductions	7 (12.7%)
Reasons for dose modification	
Hematological	5 (9.1%)
Non-hematological	2 (3.6%)
Patients with the second cycle delay >3 days	10 (18.2%)
Patients with the second cycle delay >7 days	1 (1.8%)
Reason for delay	
Adverse events	2 (3.6%)
Other*	8 (14.5%)
Median interval between first day of IC to first day of radiotherapy (IQR)	44 days (42–46)

Abbreviations: IC, induction chemotherapy; CRT, chemoradiotherapy; IQR, interquartile range.

*Other reasons for treatment delays included vacations, logistics, or personal reasons.

Supplementary Table 2. Compliance to concurrent chemoradiotherapy

Variable	IC + CRT Group (n = 55)	CRT Group (n = 55)
Patients receiving concurrent chemotherapy	55 (100%)	55 (100%)
Patients completing at least two weeks of chemotherapy during RT	55 (100%)	55 (100%)
Patients completing five weeks of chemotherapy during RT	36 (65.5%)	44 (80.0%)
Patients receiving RT	55 (100%)	55 (100%)
Patients completing RT	45 (81.8%)	50 (90.9%)
Median dose (IQR)	60 Gy (60–60)	60 Gy (60–60)
Median dose per fraction (IQR)	2.14 Gy (2.0–2.14)	2.14 Gy (2.13–2.14)
Median duration of RT (IQR)	38 days (36–41)	39 days (37–41)
Reason for premature cessation of RT		
Adverse events	7 (12.7%)	4 (7.3%)
Patient refusal	2 (3.6%)	1 (1.8%)
Investigator decision	1 (1.8%)	0 (0%)

Abbreviations: IC, induction chemotherapy; CRT, chemoradiotherapy; RT, radiotherapy; IQR, interquartile range.

Supplementary Table 3. Death reason according to treatment group

Variable	IC + CRT Group (n = 55)	CRT Group (n = 55)
Death	33 (60.0%)	35 (63.6%)
Cancer-specific	30 (54.5%)	30 (54.5%)
Non-cancer specific	3 (5.5%)	5 (9.1%)
Second primary tumor	1 (1.8%)	1 (1.8%)
Pneumonia	1 (1.8%)	2 (3.6%)
Cardiovascular diseases	1 (1.8%)	1 (1.8%)
Unknown	0 (0%)	1 (1.8%)

Abbreviations: IC, induction chemotherapy; CRT, chemoradiotherapy.

Supplementary Table 4. Adverse events during induction chemotherapy

Event	IC + CRT Group (n = 55), %			
	Grade 1	Grade 2	Grade 3	Grade 4
Anemia	38 (69.1)	2 (3.6)	0	0
Leukopenia	4 (7.3)	7 (12.7)	6 (10.9)	2 (3.6)
Neutropenia	4 (7.3)	1 (1.8)	4 (7.3)	5 (9.1)
Febrile neutropenia	0	0	2 (3.6)	0
Thrombocytopenia	4 (7.3)	2 (3.6)	0	0
Hepatotoxic event	6 (10.9)	2 (3.6)	0	0
Nephrotoxic event	3 (5.5)	1 (1.8)	0	0
Nausea/vomiting	12 (21.8)	3 (5.5)	1 (1.8)	0
Diarrhea	3 (5.5)	2 (3.6)	0	0
Weight loss	3 (5.5)	0	0	0
Fatigue	4 (7.3)	2 (3.6)	0	0

Abbreviations: IC, induction chemotherapy; CRT, chemoradiotherapy.

Supplementary Figure 1. Compliance to concurrent chemotherapy (A) and actual radiation dose (B).

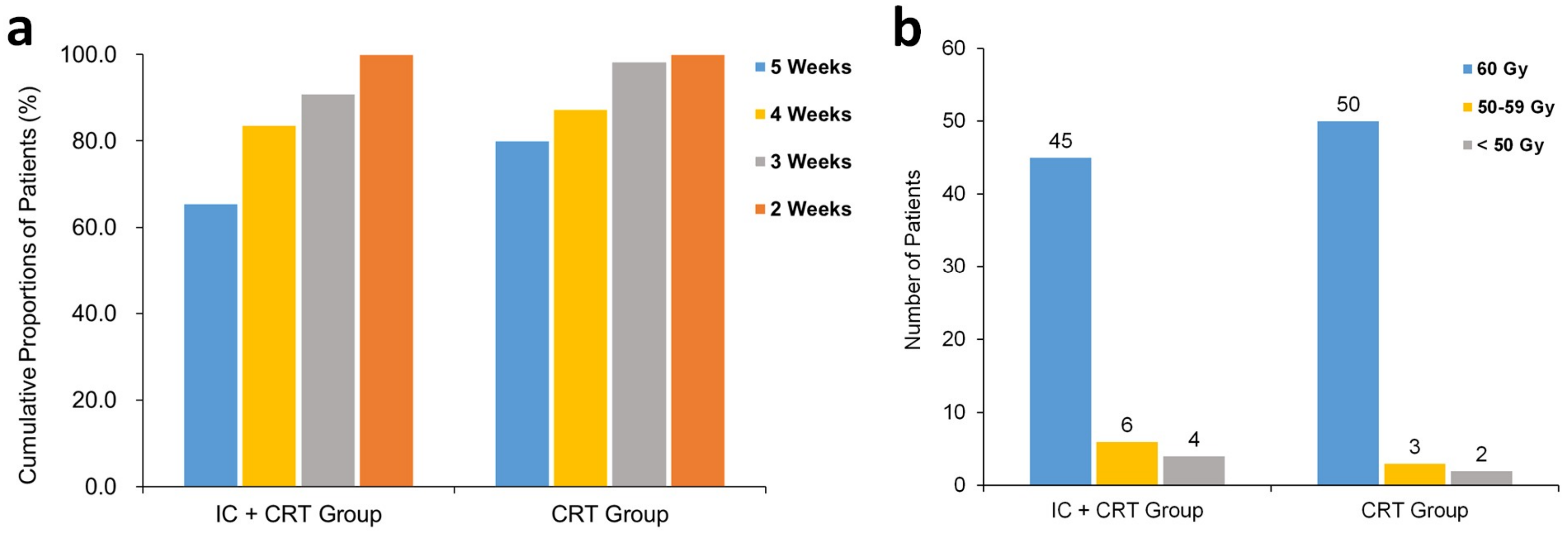

Supplementary Figure 2. Cumulative incidence of recurrences in the two treatment groups. A competing risk analysis was used (2-sided).

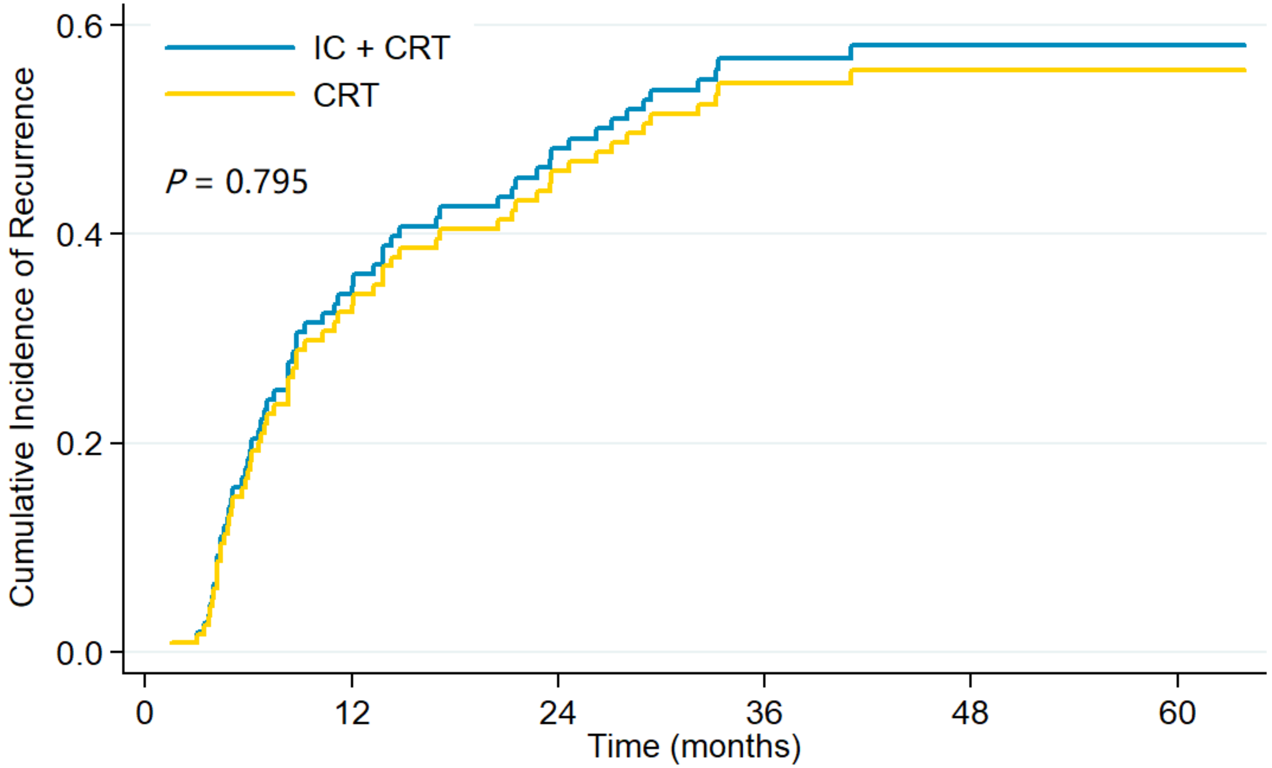

Supplement: Supplementary file 1 — Supplementary Information [file 41467_2021_24288_MOESM1_ESM.pdf]
