# Supplementary material for: Induction chemotherapy followed by definitive chemoradiotherapy versus chemoradiotherapy alone in esophageal squamous cell carcinoma: a randomized phase II trial
Source: Nat Commun. 2021 Jun 29;12:4014. doi: 10.1038/s41467-021-24288-1 (PMC8242031; doi:10.1038/s41467-021-24288-1)
Supplement: Supplementary file 3 — Description of Additional Supplementary Files [file 41467_2021_24288_MOESM3_ESM.pdf]

Description of Additional Supplementary Files

File Name: Supplementary Data 1
Description: Baseline patient information, survival outcomes, and other therapeutic information.
