# Supplementary material for: Induction chemotherapy followed by definitive chemoradiotherapy versus chemoradiotherapy alone in esophageal squamous cell carcinoma: a randomized phase II trial
Source: Nat Commun. 2021 Jun 29;12:4014. doi: 10.1038/s41467-021-24288-1 (PMC8242031; doi:10.1038/s41467-021-24288-1)
Supplement: Supplementary file 5 — Reporting Summary [file 41467_2021_24288_MOESM5_ESM.pdf]

nature research

Corresponding author(s): *Double-blind peer review submissions: write DBPR and your manuscript number here instead of author names.*

Last updated by author(s): *YYYY-MM-DD*

Reporting Summary

Nature Research wishes to improve the reproducibility of the work that we publish. This form provides structure for consistency and transparency in reporting. For further information on Nature Research policies, see our Editorial Policies and the Editorial Policy Checklist.

Statistics

For all statistical analyses, confirm that the following items are present in the figure legend, table legend, main text, or Methods section.

n/a	Confirmed	
☐	☒	The exact sample size (*n*) for each experimental group/condition, given as a discrete number and unit of measurement
☒	☐	A statement on whether measurements were taken from distinct samples or whether the same sample was measured repeatedly
☐	☒	The statistical test(s) used AND whether they are one- or two-sided<br>*Only common tests should be described solely by name; describe more complex techniques in the Methods section.*
☐	☒	A description of all covariates tested
☐	☒	A description of any assumptions or corrections, such as tests of normality and adjustment for multiple comparisons
☐	☒	A full description of the statistical parameters including central tendency (e.g. means) or other basic estimates (e.g. regression coefficient) AND variation (e.g. standard deviation) or associated estimates of uncertainty (e.g. confidence intervals)
☐	☒	For null hypothesis testing, the test statistic (e.g. *F*, *t*, *r*) with confidence intervals, effect sizes, degrees of freedom and *P* value noted<br>*Give P values as exact values whenever suitable.*
☒	☐	For Bayesian analysis, information on the choice of priors and Markov chain Monte Carlo settings
☒	☐	For hierarchical and complex designs, identification of the appropriate level for tests and full reporting of outcomes
☒	☐	Estimates of effect sizes (e.g. Cohen's *d*, Pearson's *r*), indicating how they were calculated

Our web collection on statistics for biologists contains articles on many of the points above.

Software and code

Policy information about availability of computer code

Data collection	No software was used.
Data analysis	SPSS 21.0 software (SPSS Inc., Chicago, IL) and Stata software (version 12.0).

For manuscripts utilizing custom algorithms or software that are central to the research but not yet described in published literature, software must be made available to editors and reviewers. We strongly encourage code deposition in a community repository (e.g. GitHub). See the Nature Research guidelines for submitting code & software for further information.

Data

Policy information about availability of data

All manuscripts must include a data availability statement. This statement should provide the following information, where applicable:
- Accession codes, unique identifiers, or web links for publicly available datasets
- A list of figures that have associated raw data
- A description of any restrictions on data availability

All study data are presented in the manuscript and supplementary files. Additional raw data that support the findings of this study are available from the corresponding author upon reasonable request. The data regarding the baseline patient information, survival outcomes, and other detailed therapeutic information have been deposited in the Research Data Deposit public platform (www.researchdata.org.cn).

Field-specific reporting

Please select the one below that is the best fit for your research. If you are not sure, read the appropriate sections before making your selection.

- [x] Life sciences
- [ ] Behavioural & social sciences
- [ ] Ecological, evolutionary & environmental sciences

For a reference copy of the document with all sections, see nature.com/documents/nr-reporting-summary-flat.pdf

Life sciences study design

All studies must disclose on these points even when the disclosure is negative.

Sample size	With a two-sided type I error of 0.05, a power of 80%, and a randomization ratio of 1:1, a total sample size of 98 patients would be required to demonstrate an improvement of 25% in the ORR (from 60% in the CRT group to 85% in the IC + CRT group), on the basis of previous reports. Assuming a 10% dropout rate of patients, the final sample size was 108 (54 patients per group).
Data exclusions	No date were excluded from the analyses.
Replication	NA.
Randomization	Eligible patients were randomly assigned in a 1:1 ratio in blocks of four to receive either induction chemotherapy followed by concurrent CRT (IC + CRT group) or CRT alone (CRT group) without stratification. Random assignment was conducted by a computer-generated random number code at the Clinical Trials Center of Sun Yat-sen University Cancer Center. The random allocations were contained in sequentially numbered, opaque, sealed envelopes prepared by a statistician. Patients and clinicians were not masked to treatment assignments.
Blinding	This is an open-label, randomized trial. Therefore, the investigators were not blinded to group allocation.

Reporting for specific materials, systems and methods

We require information from authors about some types of materials, experimental systems and methods used in many studies. Here, indicate whether each material, system or method listed is relevant to your study. If you are not sure if a list item applies to your research, read the appropriate section before selecting a response.

Materials & experimental systems

n/a	Involved in the study	
x		Antibodies
x		Eukaryotic cell lines
x		Palaeontology and archaeology
x		Animals and other organisms
	x	Human research participants
	x	Clinical data
x		Dual use research of concern

Methods

n/a	Involved in the study	
x		ChIP-seq
x		Flow cytometry
x		MRI-based neuroimaging

Human research participants

Policy information about studies involving human research participants

Population characteristics	Previously untreated, histologically proven squamous cell carcinoma of the thoracic esophagus; stage II to IVA according to the 6th TNM staging system of the American Joint Committee on Cancer; 18 to 70 years of age; Eastern Cooperative Oncology Group performance status of 2 or below; and adequate hematological, cardiac, pulmonary, hepatic, and renal function.
Recruitment	Eligible patients were randomly assigned in a 1:1 ratio in blocks of four to receive either induction chemotherapy followed by concurrent CRT (IC + CRT group) or CRT alone (CRT group) without stratification.
Ethics oversight	Institutional Review Board of Sun Yat-sen University Cancer Center

Note that full information on the approval of the study protocol must also be provided in the manuscript.

Clinical data

Policy information about clinical studies

All manuscripts should comply with the ICMJEguidelines for publication of clinical research and a completedCONSORT checklist must be included with all submissions.

Clinical trial registration	NCT02403531

Study protocol	The protocol was uploaded as supplementary materials.
Data collection	From May 2015 to September 2017, 126 patients were assessed and 110 were randomly assigned to the two treatment groups. The cut-off date of data collection was September 10, 2020.
Outcomes	The primary endpoint was ORR, which was defined as the proportion of patients who achieved a complete response or partial response at 3 months after the completion of CRT. The secondary endpoints were OS, defined as the time from enrollment to death or censored at the last date of follow-up; progression-free survival (PFS), defined as the time from enrollment to the date of disease progression or death from any cause or censored at the last date of follow-up; adverse events; and quality of life. Categorical variables were compared using the chi-square test or Fisher's exact test. Kaplan-Meier method was used to estimate OS and PFS, and log-rank test was used to examine survival differences between the two groups.
